# The role of mechanical pressure difference in the generation of membrane voltage under conditions of concentration polarization

**DOI:** 10.1007/s10867-016-9413-8

**Published:** 2016-04-08

**Authors:** Sławomir Grzegorczyn, Andrzej Ślęzak

**Affiliations:** Department of Biophysics, School of Medicine with the Division of Dentistry in Zabrze, Medical University of Silesia, 19 H. Jordan Str., 41808 Zabrze, Poland; Institute of Health and Nutrition Sciences, Department of Biophysics, Częstochowa University of Technology, 36B Armia Krajowa Al, 42200 Częstochowa, Poland

**Keywords:** Membrane transport, Concentration polarization, Bacterial cellulose membrane, Mechanical pressure difference, Kedem–Katchalsky equations

## Abstract

The mechanical pressure difference across the bacterial cellulose membrane located in a horizontal plane causes asymmetry of voltage measured between electrodes immersed in KCl solutions symmetrically on both sides of the membrane. For all measurements, KCl solution with lower concentration was above the membrane. In configuration of the analyzed membrane system, the concentration boundary layers (CBLs) are created only by molecular diffusion. The voltages measured in the membrane system in concentration polarization conditions were compared with suitable voltages obtained from the model of diffusion through CBLs and ion transport through the membrane. An increase of difference of mechanical pressure across the membrane directed as a difference of osmotic pressure always causes a decrease of voltage between the electrodes in the membrane system. In turn, for mechanical pressure difference across the membrane directed in an opposite direction to the difference of osmotic pressure, a peak in the voltage as a function of mechanical pressure difference is observed. An increase of osmotic pressure difference across the membrane at the initial moment causes an increase of the maximal value of the observed peak and a shift of this peak position in the direction of higher values of the mechanical pressure differences across the membrane.

## Introduction

The bacterial cellulose membranes, similar to other polymer membranes, undergo relatively strong concentration polarization [1, 2] and they are therefore convenient materials for study of diffusive and convective processes near membrane areas [3–6]. One of the many important applications of bacterial cellulose are membrane dressings on difficult-to-heal wounds [7–9]. Due to the properties of cellulose fiber structure [10], membrane dressings allow generating an appropriate microenvironment near the membrane, leading to the acceleration of the healing process [11, 12]. Studies of nanocomposite membranes of bacterial cellulose reveal interesting new properties of these materials and their applications [13–15]. In case of membranes and membrane dressings, concentration polarization is an important phenomenon significantly limiting the transport of volumes and solutes through the membranes, influencing solute fluxes through the membrane [16, 17] as well as the measured voltages and currents through the membrane in the case of transport of electrolyte solutions [18–21]. A characteristic feature of concentration polarization is concentration boundary layers (CBLs) [16, 22, 23] with concentration profiles dependent on the initial conditions [16, 24], type of solute, properties of the membrane itself, and the membrane orientation in relation to the direction of the gravitational field vector [22, 25–27]. In case of membranes arranged in a horizontal plane, diffusion and hydrodynamic instability of CBLs are the main phenomena that influence the CBL thickness [21, 28, 29] and can be visualized by interferometric methods [30]. Furthermore, the mechanical pressure difference (∆*P*) across the membrane and volume flux (*J*_*v*_) through the membrane cause changes in thickness of the CBLs [31] and distribution of solute near the membrane [32]. In addition, it can be expected that fixing ∆*P* across the membrane different from zero will cause a significant change in volume and solutes fluxes through the membrane, influencing concentrations in CBLs near the membrane. Changes of CBLs caused by pressure difference across the membrane were visualized by interferometric methods [33]. ∆*P* across the membrane is the main thermodynamic force used in reverse osmosis [34–36] and pressure retarded osmosis [37–40]. For some membranes, ∆*P* across the membrane can cause asymmetry of volume fluxes [41].

Previous studies of voltages between electrodes dipped in solutions in the membrane system were carried out with zero mechanical pressure difference across the membrane. The procedure of measurement of voltages between the Ag|AgCl electrodes placed in the solutions on both sides of the membrane was described in a previous paper [23]. It was found that concentration and time characteristics of voltages measured in the membrane system depend on system configuration and the initial concentrations in chambers of the membrane system [23, 42]. In this article, the influence of pressure difference across the membrane on the voltage characteristics measured in the membrane system are shown in conditions of concentration polarization of membrane. Additionally, the characteristics of changes of ion concentrations near the membrane during evolution of CBLs disturbed by Δ*P* fixed on the membrane were calculated. The measurements were carried out in the membrane system with bacterial cellulose membrane located in the horizontal plane and aqueous KCl solutions. The bacterial cellulose membrane is an electroneutral membrane and does not contain bound ions. Moreover, the mathematical model of transport of ions through the membrane and CBLs was elaborated on the basis of the partial differential diffusion equation for transport of ions in CBLs and Kedem-Katchalsky equations for membrane transport. This model was verified on the basis of experimental data.

## Theory

Electrolyte transport through biological and artificial membranes can be described by the Kedem–Katchalsky equations derived from irreversible thermodynamics. For homogeneous and dilute electrolyte solutions (for example single-salt system), these equations can be written in the form [43]

1 $$ {J}_v={L}_p\left(\varDelta P-{\displaystyle \sum_{j=1}^n{\sigma}_j\kern0.1em \varDelta {\pi}_j}-\beta i\right) $$

2 $$ {J}_s={\overline{C}}_s\left(1-{\sigma}_s\right){J}_v+{\displaystyle \sum_{j=1}^n{\omega}_{sj}\varDelta {\pi}_j}+\frac{t_s}{z_sF}i $$

where *J*_*v*_ and *J*_*s*_ are the volume and ion fluxes, where the *s* indicates the ion of interest, which is present with *n* ions in solution, *i* represents the current density through the membrane, *ΔP* = *P*_*h*_ − *P*_*l*_ is the hydrostatic pressure difference on the membrane, *Δπ*_*s*_ = *RT*(*C*_*h*_ − *C*_*l*_) is the osmotic pressure difference, ${\overline{C}}_s=\left({C}_h-{C}_l\right){\left[ \ln \left({C}_h{C_l}^{-1}\right)\right]}^{-1}$ is the average ion concentration in the membrane. Furthermore, *C*_*h*_ and *C*_*l*_ (*C*_*h*_ > *C*_*l*_) are the ion concentrations, *L*_*p*_ is the hydraulic permeability coefficient of the membrane, *σ*_*s*_ is reflection coefficient, *ω*_*s*_ is the permeability coefficient, *β* – electroosmotic coefficient, *t*_*s*_ – transference number of ion *s*, *F* – Faraday number, *R* – gas constant and *T* – absolute temperature. Due to the lack of accumulation or depletion of ions near (and in) the electroneutral membrane and because of the electroneutrality of solutions, it can be concluded that *J*_+_ = *J*_−_ = *J*_KCl_ [43, 44]. For this reason, in the vicinity of the electroneutral membrane, there is only a phenomenon of concentration polarization of the membrane having an important influence on KCl transport through the membrane.

Substituting Eq. (1) into Eq. (2), the total flux of suitable ions (*s*) through the membrane can be written as

3 $$ {J}_s=\left(1-{\sigma}_s\right){\overline{C}}_s{L}_p\varDelta P+{\displaystyle \sum_{j=1}^n\left({\omega}_{sj}-{\overline{C}}_s\left(1-{\sigma}_s\right){\sigma}_j{L}_p\right)\varDelta {\pi}_j}+i\left[\frac{t_s}{z_sF}-{\overline{C}}_s\left(1-{\sigma}_s\right){L}_p\beta \right] $$

Due to the very high internal resistance of the meter (0.1 GΩ) and the electroneutrality of electrolyte solutions, the current density through the membrane (electroneutral membrane without bounded ions) during the measurements is negligible. Therefore, the third term in Eq. (3) is negligibly small, which means that Eq. (3) can be simplified to

4 $$ {J}_s=\left(1-{\sigma}_s\right){\overline{C}}_s{L}_p\varDelta P+{\displaystyle \sum_{j=1}^n\left({\omega}_{sj}-{\overline{C}}_s\left(1-{\sigma}_s\right){\sigma}_j{L}_p\right)\varDelta {\pi}_j} $$

For a single-salt system (KCl) (*n* = 2, s = + or -), it can be assumed that nondiagonal coefficients are much lower than diagonal coefficients ${\omega}_{sj}-{\overline{C}}_s\left(1-{\sigma}_s\right){\sigma}_j{L}_p<<{\omega}_{ss}-{\overline{C}}_s\left(1-{\sigma}_s\right){\sigma}_s{L}_p\ \mathrm{f}\mathrm{o}\mathrm{r}\kern0.30em s\ne j$) [43], so Eq. (4) can be written as

5 $$ {J}_s=\left(1-{\sigma}_s\right){\overline{C}}_s{L}_p\varDelta P+\left({\omega}_{ss}-{\overline{C}}_s\left(1-{\sigma}_s\right){\sigma}_s{L}_p\right)\varDelta {\pi}_s $$

This form of ion flux through the membrane depends on the membrane thermodynamic forces: the mechanical pressure difference (Δ*P*) and the osmotic pressure difference across the membrane (Δ*π*_*s*_). Ion flux through the membrane, described by Eq. (5), causes a disturbance of ion concentrations on both sides of the membrane and the diffusion in near-membrane areas leads to rebuilding of CBLs at membrane surfaces. In addition, the volume flux through the membrane and through the chambers is associated with the movement of the solution and depends mainly on the pressure difference across the membrane. Taking into account the volume flux through the membrane, causing an additional movement of solution in CBLs and membrane (in direction *x*, perpendicular to membrane surface), the concentration changes of suitable ions in CBLs can be described by the equation

6 $$ \frac{\partial C}{\partial t}=-\frac{\partial {J}_s}{\partial x}={D}_s\frac{\partial^2C}{\partial {x}^2}-v\frac{\partial C}{\partial x} $$

where *D*_*s*_ is the diffusion coefficient of ions *s* in solution and *v* is velocity of solution caused by pressure difference across the membrane. Due to continuity law of flow, this velocity is equal to volume flux through the membrane in the case when the surface of the membrane is equal to the cross-sectional area of chambers.

In order to determine the change in time of concentrations in CBLs, Eq. (5) was used to calculate the flux of ions through the membrane, while Eq. (6) was written in difference form (Appendix), with assumption that velocity of solution through the membrane and chambers is related to the pressure difference across the membrane. To simplify the model, it was assumed that the surface of the membrane is equal to the cross-sectional area of chambers containing solution. The area near the membrane was divided into *N* layers parallel to the membrane surface and having a thickness *d*_*w*_ each. Accordingly, the difference equations for layers adjacent to the membrane surfaces (*n *= 1) can be presented on the basis of Eqs. (5) and (6) for the chamber with lower concentration (the chamber over the membrane) as Eq. (7) and for the chamber with higher concentration (the chamber under the membrane) as Eq. (8):

7 $$ {C}_{l,1}^{k+1}={C}_{l,1}^k+\frac{\varDelta t}{d_w}\left({A}^k+{B}^kRT\left({C}_{h,1}^k-{C}_{l,1}^k\right)\right)-\frac{\varDelta t}{d_w^2}{D}_s\left({C}_{l,1}^k-{C}_{l,2}^k\right)-\frac{\varDelta t}{d_w}\left({C}_{l,1}^k-{C}_{l,2}^k\right){L}_p\varDelta P $$

8 $$ {C}_{h,1}^{k+1}={C}_{h,1}^k-\frac{\varDelta t}{d_w}\left({A}^k+{B}^kRT\left({C}_{h,1}^k-{C}_{l,1}^k\right)\right)-\frac{\varDelta t}{d_w^2}{D}_s\left({C}_{h,1}^k-{C}_{h,2}^k\right)-\frac{\varDelta t}{d_w}\left({C}_{h,1}^k-{C}_{l,2}^k\right){L}_p\varDelta P $$

where *C*_*l*,1_^*k*^, *C*_*h*,1_^*k*^ are the concentrations in layers adjacent to the membrane (second subscript *n *= *1*) suitably in the chamber with lower concentration (first subscript *l)* and in the chamber with higher concentration (first subscript *h*) at time point *k*, ${\overline{C}}^k=\left({C}_{h,1}^k-{C}_{l,1}^k\right)\cdot {\left[ \ln \left({C}_{h,1}^k{\left({C}_{l,1}^k\right)}^{-1}\right)\right]}^{-1}$ is the average concentration in the membrane and ${A}^k=\left(1-{\sigma}_s\right){\overline{C}}^k\;{L}_p\varDelta P$, ${B}^k={\omega}_s-{\sigma}_s{L}_p\left(1-{\sigma}_s\right){\overline{C}}^k$. Moreover, *d*_*w*_ is the thickness of the layer and Δ*t* is a time interval used in the recursive method of solution of differential equations. It was assumed that the velocity of solution movement in chambers is equal to volume flux through the membrane *v* = *J*_*vk*_ = *L*_*p*_*ΔP*. In turn, the difference equations for concentrations in other layers of CBLs (for *n* > 1) obtained on the basis of Eq. (6) take the form of Eq. (9) for layers above the membrane (chamber with lower solute concentration) and of Eq. (10) for layers under the membrane (chamber with higher solute concentration) as follows:

9 $$ {C}_{l,n}^{k+1}={C}_{l,n}^k+\frac{\varDelta t}{d_w^2}{D}_s\left({C}_{l,n+1}^k+{C}_{l,n-1}^k-2{C}_{l,n}^k\right)-\frac{\varDelta t}{d_w}\left({C}_{l,n}^k-{C}_{l,n-1}^k\right){L}_p\varDelta P, $$

10 $$ {C}_{h,n}^{k+1}={C}_{h,n}^k+\frac{\varDelta t}{d_w^2}{D}_s\left({C}_{h,n+1}^k+{C}_{h,n-1}^k-2{C}_{h,n}^k\right)+\frac{\varDelta t}{d_w}\left({C}_{h,n}^k-{C}_{h,n-1}^k\right){L}_p\varDelta P. $$

Numerical solutions of differential Eqs. (7–10) allow determination of the changes of concentrations in time at any point in the CBLs. Eqs. (5) and (7–10) have been used to obtain quantitative characteristics of the membrane system with the bacterial cellulose membrane and hydrodynamically stable configuration with lower KCl concentration in the chamber above the membrane. In such a system, CBLs are reconstructed only by diffusion. The mechanical pressure difference across the membrane was stabilized during measurements of voltage between electrodes.

The voltage between Ag|AgCl electrodes placed in KCl solutions on both sides of the membrane can be written in the form [24]

11 $$ \varDelta {\psi}^k=-\frac{2RT}{F}\left[\left({\overline{t}}_{+}-{t}_{+}\right) \ln \left(\frac{\gamma_{h,1}^k{C}_{h,1}^k}{\gamma_{l,1}^k{C}_{l,1}^k}\right)+{t}_{+} \ln \left(\frac{\gamma_{h,n\hbox{'}}^{*k}{C}_{h,n\hbox{'}}^{*k}}{\gamma_{l,n\hbox{'}\hbox{'}}^{*k}{C}_{l,n\hbox{'}\hbox{'}}^{*k}}\right)\right] $$

where *γ*_*h*,1_^*k*^*C*_*h*,1_^*k*^, *γ*_*l*,1_^*k*^*C*_*l*,1_^*k*^ and *γ*_*h*,*n* '_^* *k*^*C*_*h*,*n* '_^* *k*^_,_*γ*_*l*,*n* ' '_^* *k*^*C*_*l*,*n* ' '_^* *k*^ are the products of ion activity coefficients and concentrations at the membrane surfaces and at the electrode surfaces (marked with an asterisk) placed on both sides of the membrane, *n’* and *n”* are the numbers of layers where the electrodes are placed. Besides, *t*_*+*_ and ${\overline{t}}_{+}$ are the apparent transference numbers for K^+^ ions in solution and in the membrane, and *R*, *T*, and *F* are the gas constant, thermodynamic temperature, and Faraday constant, respectively. Solutions in chambers are homogeneous during mechanical stirring, so for all *n C*_*h*,n_^* 0^ = *C*_*h*_ and *C*_*l*,n_^* 0^ = *C*_*l*_. Turning off mechanical stirring of solutions is the initial moment of measurement of voltage in the membrane system so the conditions *C*_*h*,n_^* 0^ = *C*_*h*_ and *C*_*l*,n_^* 0^ = *C*_*l*_ for all *n* are the initial conditions for calculations in the model based on Eqs. (7–10). After turning off the mechanical stirring, the solutions near the membrane became non-homogeneous and concentrations near membrane surfaces fulfill the conditions *C*_*h*_*≥ C*_*h*,*n* ' >1_^* *t* ≠ 0^*> C*_*h*,1_^*t* ≠ 0^*> C*_*l*,1_^*t* ≠ 0^*> C*_*l*,*n* ' ' >1_^* *t* ≠ 0^*≥ C*_*l*_. The concentration polarization of the membrane causes that in steady state of the membrane system the concentrations on both sides of the membrane at its surfaces are almost equal. This also causes that the ion transference numbers in membrane and in solution tend to similar values and the osmotic pressure difference across the membrane quickly (in several minutes) tends to a small value, close to zero, in comparison to the initial osmotic pressure difference. For this reason, the first component in Eq. (11) can be taken as equal to zero, which means that the voltage between the electrodes in the steady state (*k*=*st*) can be written as

12 $$ {\left(\varDelta \psi \right)}^{k=st}=\frac{2RT}{F}\left[{t}_{+} \ln \left(\frac{\gamma_{h,n\hbox{'}}^{*k=st}{C}_{h,n\hbox{'}}^{*k=st}}{\gamma_{l,n\hbox{'}\hbox{'}}^{*k=st}{C}_{l,n\hbox{'}\hbox{'}}^{*k=st}}\right)\right]. $$

Indices *n’* and *n”* refer to layers with electrodes in chambers with higher and lower concentration of solutions, respectively. Therefore, using the voltage between electrodes measured in steady state defined by Eq. (12), the ratio of ion activities at surfaces of electrodes in steady states can be calculated and presented in the form

13 $$ \frac{a_i^{*}}{a_e^{*}}=\frac{\gamma_{h,n\hbox{'}}^{*k=st}{C}_{h,n\hbox{'}}^{*k=st}}{\gamma_{l,n\hbox{'}\hbox{'}}^{*k=st}{C}_{l,n\hbox{'}\hbox{'}}^{*k=st}}= \exp \left[\frac{F{\left(\varDelta \psi \right)}^{k=st}}{2RT\;{t}_{+}}\right], $$

where *a*_i_^*^ and *a*_e_^*^ are activities of K^+^ ions near electrodes in solutions under and above the membrane, respectively.

## Experiment

**Fig. 1:**
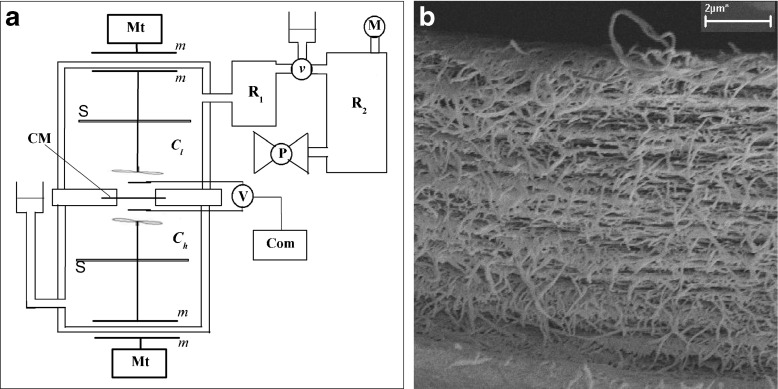
**a** The measurement system: membrane (CM), motor (Mt) and magnets (*m*) of stirring system, millivoltmeter (V) connected with Ag|AgCl electrodes and computer (Com), pump (P), manometer (M), valve (ν), *C*_*h*_, *C*_*l*_ are the KCl concentrations, homogeneous in whole chambers at initial moment, S is the support to the stirring system, R_1_ and R_2_ are reservoirs filled suitably with a solution and with air. **b** Electron microscope image of a cross section of the bacterial cellulose membrane (CM), obtained using a Zeiss Supra 35 with magnification of 15000×

The measurements were carried out in the membrane system presented in Fig. 1a with bacterial cellulose membrane (*Biofill*), oriented in horizontal plane, between two chambers with the volume of 1.75 × 10^−4^ m^3^ each, filled with aqueous solutions of KCl (*C*_*l*_ < *C*_*h*_). The membrane system includes two reservoirs stabilizing pressure difference across the membrane (R_1_ filled with a solution such as in the chamber above the membrane and R_2_ filled with air). Valve ν is opened to the upper and left vessels during filling of the chambers with solutions. Next, the valve ν is switched to open for left and right vessels (upper vessel is locked) and the pressure in R_1_ , R_2_ and in membrane system was fixed by means of pump (P) and controlled by a manometer M. The reservoir R_2_ (V_2_ = 5 dm^3^) allows stabilizing the pressure across the membrane during measurement of voltage in the membrane system. After fixing the pressure in the membrane system, the stirring of solutions was turned off, and simultaneously the measurement of voltage was begun.

The transport properties of bacterial cellulose membrane (*Biofill*) and KCl solutions are determined by the following coefficients: diffusion permeability coefficient *ω*_*s*_ = 1.94 × 10^-9^ mol N^−1^ s^−1^, the hydraulic permeability coefficient *L*_*p*_ = 6.5 × 10^−11^ m^3^ N^−1^ s^−1^ and reflection coefficient *σ* = 0.0034. Values of these coefficients were measured experimentally by methods described in [42]. An electron microscope image of a cross section of the bacterial cellulose membrane is presented in Fig. 1b.

At the initial moment, the chamber under the membrane contained aqueous KCl solution with higher concentration *C*_*h*_, and chamber over the membrane contained aqueous KCl solution with lower concentration *C*_*l*_. This configuration of the membrane system is hydrodynamically stable, which means that CBLs near the membrane are reconstructed only by diffusion, because layers of solution in CBL with lower density are over layers of solution with higher density. At the initial moment in each experiment, the lower KCl concentration amounted to *C*_*l*_ = 0.01 mol m^−3^, while the concentration *C*_*h*_ was changed in the range from 0.5 mol m^−3^ to 10 mol m^−3^. The area of the membrane under investigation was 3.1 × 10^−4^ m^2^. In order to assure homogeneity of solutions at the initial moment, mechanical stirring was used with a rate of 500 rpm. Turning off stirring of solutions is the beginning of the measurement of voltage between the electrodes in the membrane system. The difference of mechanical pressure (Δ*P*) fixed on the membrane during measurements of voltage between electrodes in the membrane system was changed in the range from Δ*P* = −50 kPa to Δ*P* = +20 kPa and was stabilized during measurements. When higher pressure was applied to the chamber with lower KCl concentration, Δ*P* on the membrane was treated as negative because Δ*P* on the membrane was oppositely directed to the osmotic pressure difference.

Time characteristics of voltage between electrodes after turning off mechanical stirring of solutions in the membrane system were measured by means of Ag|AgCl electrodes. The electrodes were located on both sides of the membrane at the same distance from the membrane surfaces: *d* = 6 mm. Voltage between electrodes was measured by means of voltmeter (MERATRONIK Type U726) connected to a computer. The input impedance of the millivoltmeter amounted to 0.1 GΩ, and its accuracy was 0.1 mV. The membrane system was thermostated and enclosed by a Faraday cage, in order to prevent electrical interference. The temperature of solutions was 295 ± 0.5 K. The chambers of the membrane system were filled with solutions, stirred by magnetic stirrers as long as the voltage was settled, no longer than 1–2 min. After turning off mechanical stirring, the voltage was measured every 2 s during 100 min. The error of preparation of solution concentrations was lower than 1.5%, while relative error of voltage measurements for this same initial condition was lower than 5%.

## Numerical calculations

On the basis of finite-difference Eqs. (7–10) resulting from partial differential equation of diffusion with solution movement (6) and Eq. (5) for transport of K^+^ ions through the membrane, numerical calculations of concentrations in selected points near the membrane were performed by means of MathCad Prime 3.0. Then, concentrations (*C*_i_) calculated from the model have been converted into activities of K^+^ ions (*a*_i_) according to equation [45]

14 $$ {a}_i={\gamma}_i{C}_i={10}^{-\frac{A{z}_i^2\sqrt{I}}{1+ aB\sqrt{I}}}{C}_i $$

where $I=0.5\cdot {\displaystyle \sum_i{C}_i{z}_i^2}$ is the ionic force, *A* = 0.5066 and *aB* ≅ 0.99 at 295 K. The following parameter values were assumed: layer thickness *d*_*w*_ = 10^-4^ m, the time interval Δ*t* = 1s, the diffusion coefficient of KCl in aqueous solution *D*_*s*_*=* 2.01 × 10^−9^ m^2^ s^−1^, *t*_*+*_ = 0.49 transference number for K^+^ ions in aqueous solutions, *F* = 96500 C mol^−1^, *R* = 8.31 J mol^-1^ K^-1^, *L*_*p*_ = 6.5 × 10^-11^ m^3^ N^−1^ s^−1^ and *σ* = 0.0034. Unlike the experimental system, it was assumed in the model that the membrane surface is equal to the cross-sectional area of the chambers with solutions. It was also assumed that at the initial moment solutes in chambers are homogeneous, so for all layers the initial conditions: *C*_*l*,*n*_^0^ = *C*_*l*_ and *C*_*h*,*n*_^0^ = *C*_*h*_, for all *n* were fulfilled.

During solute transport through the membrane, CBLs are formed near the membrane and therefore it is important to determine the concentration distribution in the vicinity of the membrane and electrodes. Equations (5) and (7–10) allow calculation of the temporal changes of concentrations at selected distances from the membrane, i.e., at surfaces of the membrane (0 mm) and at electrode surfaces (6 mm from the surface of the membrane). At the initial moment of voltage measurements it was assumed that in the entire upper chamber (above the membrane) there was KCl solution with lower concentration equal to *C*_*l*_ = 0.01 mol m^−3^, whereas in the chamber under the membrane there was a KCl solution with a greater concentration. The preparation of the membrane system for measurement caused a disturbance of initial concentrations (mainly in the chamber with a lower concentration) so it was taken into consideration in the model in order to estimate the real concentrations in chambers at the initial moment. In order to match the results of the model [Eqs. (5) and (7–10)] to voltages measured experimentally, corrected initial KCl concentrations in the chamber over the membrane have been calculated. For this purpose, the maxima of the pressure characteristics of voltage between electrodes in steady states (Δ*ψ*_st_), measured in the membrane system (points in Fig. 5) were compared with corresponding maxima of the pressure characteristics of Δ*ψ*_st_ obtained from the calculations (solid lines in Fig. 5). As results from correction (for three values of *C*_*h*_ / *C*_*l*_ : 100, 500, and 1000) the dependence of corrected KCl concentration in the chamber over the membrane as a function of initial concentration in the chamber under the membrane may be presented as linear function

15 $$ {\left({C}_l\right)}_{\mathrm{corr}} = 7.59\kern0.5em \times \kern0.5em {10}^{-3}{C}_h + 1.985\kern0.5em \times \kern0.5em {10}^{-2}\mathrm{mol}\ {\mathrm{m}}^{-3} $$

with matching coefficient *R*^2^ = 0.972. The units of (*C*_*l*_)_corr_ and *C*_*h*_ in Eq. (15) are: mol m^−3^.

## Results and discussion

**Fig. 2:**
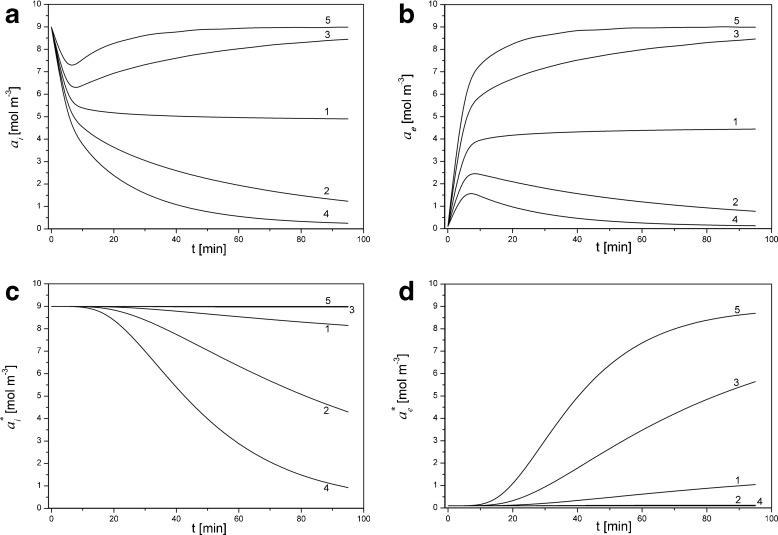
Activities of K^+^ ions at surfaces of the membrane (*a*_*i*_ , *a*_*e*_) and at surfaces of electrodes (*a*_i_^*^ , *a*_e_^*^) as functions of time for pressure differences on the membrane: 0 kPa (1), −20 kPa (2), +20 kPa (3), −40 kPa (4), and +40 kPa (5), *C*_*h*_ = 10 mol m^−3^ and (*C*_*l*_)_corr_ = 0.0957 mol m^−3^

In Fig. 2, time characteristics of activities of K^+^ ions calculated from the model (with correction of the initial concentration in chamber over the membrane) are shown for fixed pressure differences on the membrane 0 kPa (1), −20 kPa (2), +20 kPa (3), −40 kPa (4), and +40 kPa (5), where *a*_*i*_ , *a*_*e*_ are K^+^ ion activities at surfaces of the membrane (Fig. 2a and b) and *a*_i_^*^ ,  *a*_e_^*^ are K^+^ ions activities at surfaces of electrodes (6 mm from the membrane surfaces – Fig. 2c and d)) suitably in the chamber with lower concentration (subscript *e*) and higher concentration (subscript *i*). Calculations were performed for KCl concentration under the membrane at the initial moment equal to *C*_*h*_ = 10 mol m^-3^ and for the corrected initial value of KCl concentration in the chamber above the membrane, (*C*_*l*_)_corr_ = 0.0957 mol m^−3^.

As follows from the graphs in Fig. 2, turning off stirring of the solutions (*t* = 0) is the beginning of CBL reconstruction, which causes changes in time of activities of K^+^ ions at the membrane and surfaces of electrodes. After about 10 min from the initial moment for Δ*P* = 0 kPa (curves 1 in Fig. 2a and b), the activities of K^+^ ions at surfaces on both sides of the membrane (*a*_*i*_, *a*_*e*_) differ little from each other. This determines the fact that after switching off the stirring of solutions, osmotic pressure on the membrane, corresponding to the difference of solute concentrations (*C*_i_ − *C*_e_), decreases within the first few minutes to a small value in comparison with its initial value. In turn, the application of Δ*P* different from 0 causes a change of the time characteristics of ion activities. For positive pressure difference (applied in accordance with direction of osmotic pressure difference – curves 3 and 5 in Fig. 2a and b), time characteristics of K^+^ ion activities are shifted to higher values and for negative pressure difference (curves 2 and 4 in Fig. 2a and b) to smaller values in comparison to characteristics obtained without pressure difference on the membrane (curve 1 in Fig. 2a and b). This shift is greater when the pressure difference across the membrane is higher. In addition, for greater distance of points from the membrane (curves in Fig. 2c and d), the changes of activities of K^+^ ions in time are smaller.

**Fig. 3:**
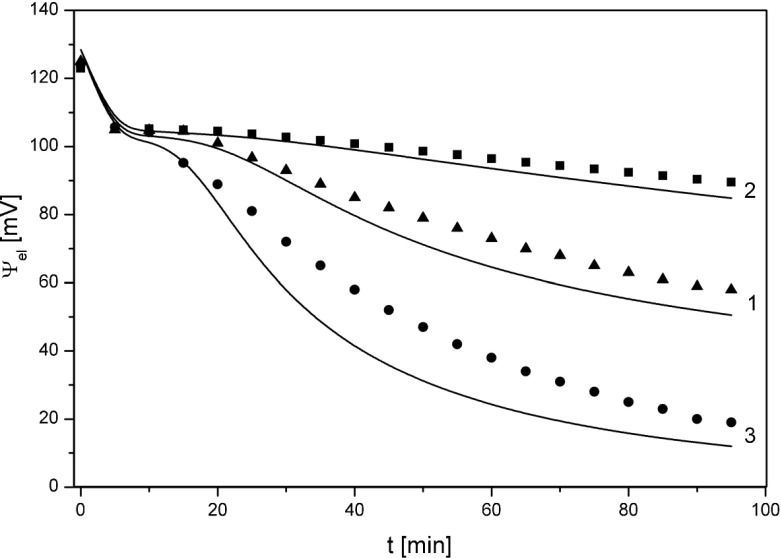
Time characteristics of voltage between electrodes (Δψ_el_) for pressure differences on the membrane: Δ*P* = 0 kPa (1), Δ*P* = −20 kPa (2) and Δ*P* = +20 kPa (3) (points – experiment, lines – model) for *C*_*h*_ = 10 mol m^−3^ and (*C*_*l*_)_corr_ = 0.0957 mol m^−3^

On the bases of time characteristics of activities of K^+^ ions at the surfaces of the membrane and electrodes presented in Fig. 2a-d and Eq. (11) the voltage between electrodes was calculated and is presented in Fig. 3 for fixed pressure differences on the membrane: Δ*P* = 0 (1), Δ*P* = - 20 kPa (2) and Δ*P* = + 20 kPa (3) (lines – calculated from the model, points - measured in experiment). Calculations have been performed for *C*_*h*_ = 10 mol m^-3^ and for the corrected initial value of KCl concentrations in the chamber above the membrane, (*C*_*l*_)_corr_ = 0.0957 mol m^−3^.

According to the results presented in Fig. 3, the character of temporal changes of voltages obtained from the model (lines) and experiment (points) are similar. The differences in the values of voltages may result from simplifications in the model such the assumption that the surface of the membrane is equal to the cross-sectional area of the chambers and neglecting the presence of the stirring system in the chambers of the measurement system.

**Fig. 4:**
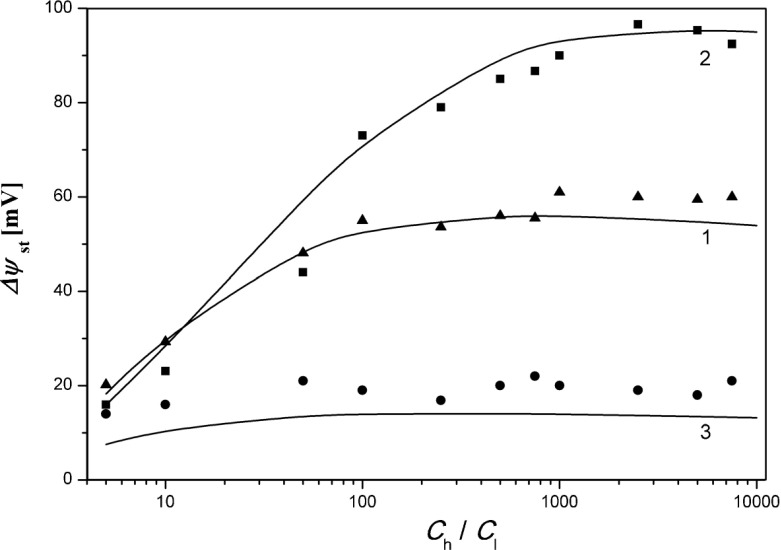
The dependencies Δ*ψ*_st_ = *f* (*C*_*h*_/*C*_*l*_) in steady states of the membrane system for fixed pressure difference across the membrane Δ*P* = 0 kPa (1), −20kPa (2), and +20kPa (3). Experimental results (*points*) and simulation results with correction (*solid lines*) are shown for KCl concentrations in chamber over the membrane

The next characteristics shown in Fig. 4 are the voltages between electrodes in steady states (100 min after turning off stirring of the solutions) as functions of the ratio of the initial concentrations in chambers Δ*ψ*_st_ = *f*(*C*_*h*_/*C*_*l*_), for mechanical pressure difference across the membrane: -20 kPa, 0 kPa, +20 kPa, (points – experiment, lines – model). Δ*ψ*_st_ was calculated from the model on the basis of Eqs. (5), (7–10), and (12), with corrected initial value of KCl concentrations in chamber above the membrane on the basis of Eq. (15).

As is apparent from the comparison of data presented in Fig. 4, agreement of voltages between electrodes obtained from the experiment and from the model in steady states as functions of the ratio of initial concentrations in the chambers is satisfactory. An increase of *C*_*h*_/*C*_*l*_ for the membrane system without difference of mechanical pressure across the membrane causes an increase of Δ*ψ*_st_ and then fixing of Δ*ψ*_st_ for *C*_*h*_/*C*_*l*_ greater than 100. The use of pressure difference across the membrane in opposite direction to osmotic pressure (curve 2) causes voltages between electrodes in steady states to be greater than in the case of Δ*P* = 0 kPa. For Δ*P* = -20 kPa and *C*_*h*_*/C*_*l*_ > 1000 values of Δ*ψ*_st_ are about 90 mV. Applying pressure difference across the membrane directed as osmotic pressure (curve 3) causes calculated Δ*ψ*_st_ to be equal to about 10 mV over the whole range of *C*_*h*_*/C*_*l*_, while values of Δ*ψ*_st_ measured in the membrane system are slightly greater, but still lower than 20 mV. From the results presented in Fig. 4 it can be stated that an increase of *C*_*h*_*/C*_*l*_ causes greater asymmetry of Δ*ψ*_st_ because of the direction of applied pressure difference across the membrane. Additionally, the dependence Δ*ψ*_st_ = *f*(*C*_*h*_*/C*_*l*_) for higher values of *C*_*h*_*/C*_*l*_ is nearly constant.

**Fig. 5:**
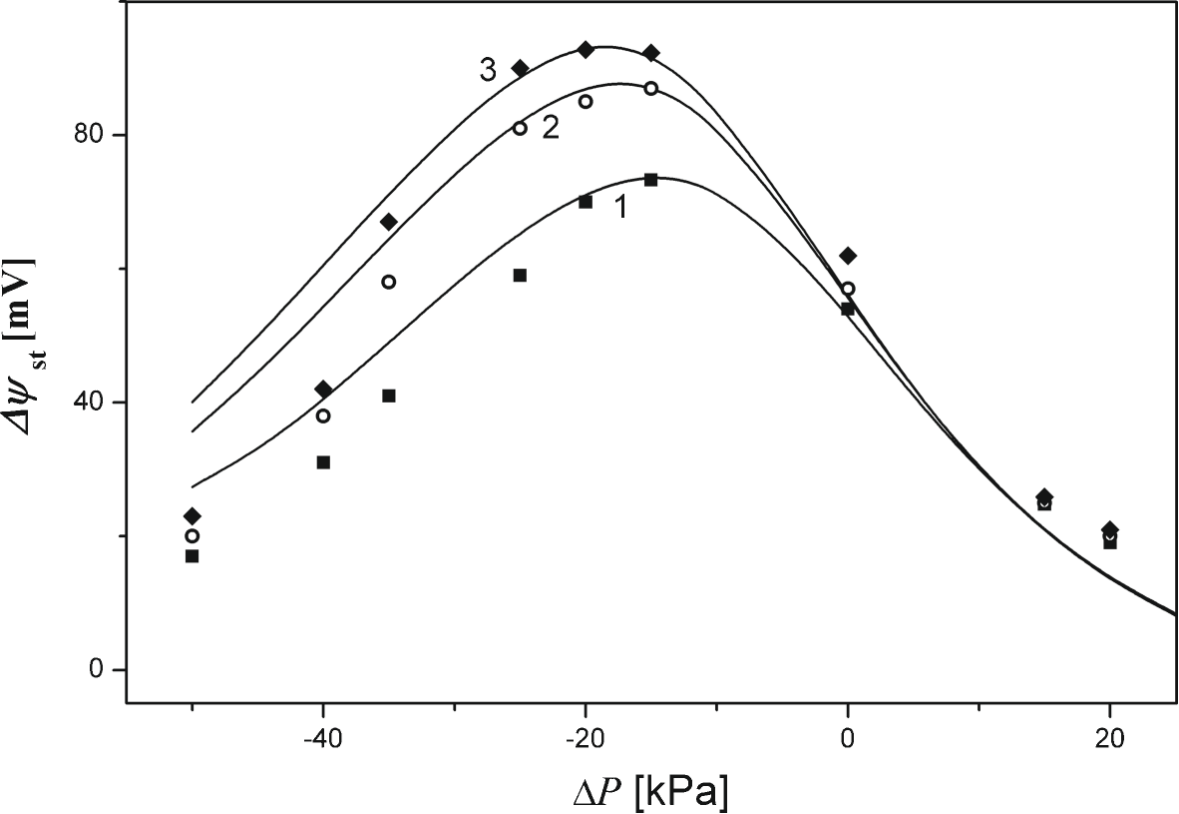
The voltage between electrodes in steady states (Δ*ψ*_st_) of the membrane system as a function of pressure difference (Δ*P*) on the membrane for experimental results (*points*) and for results from the model with correction (*solid lines*), for *C*_*h*_ */C*_*l*_ = 100 (1), 500 (2), and 1000 (3)

In Fig. 5, the pressure characteristics of voltages between electrodes in steady states are shown for *C*_*h*_*/C*_*l*_ = 100, 500, and 1000. The graphs were obtained from experiment (points) and calculated on the basis of the model of layers in CBLs with corrected initial values of KCl concentrations in the chamber above the membrane (solid lines).

**Fig. 6:**
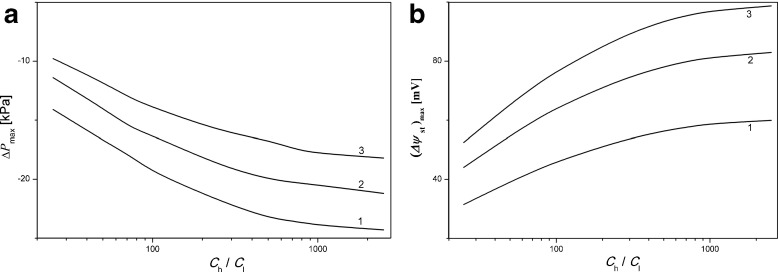
The pressure difference across the membrane at which voltage between electrodes is maximal (Δ*P*)_max_ (**a**) and maximal values of voltage in steady states (Δ*ψ*_st_)max (**b**) as functions of quotient of KCl concentrations in chambers at the initial moment, for distances of electrodes from the membrane 3 mm (1), 5 mm (2), and 7 mm (3)

Both results from the experiment (points) and from the model (lines) show that these dependencies are characterized by the appearance of maximal value of Δ*ψ*_st_ in the range of negative pressure difference across the membrane. An increase of *C*_*h*_*/C*_*l*_ on the membrane causes the height of the peak to be greater and the position of the peak to be shifted towards more negative pressure difference across the membrane. This can be associated with the higher values of osmotic pressure on the membrane for higher values of *C*_*h*_*/C*_*l*_. Therefore, to balance greater osmotic pressure across the membrane, we can expect the location of the peak to shift toward larger values of mechanical pressure differences applied in the opposite direction to the osmotic pressure. The peak position and height of the peak as functions of the ratio of concentrations in chambers at the initial moment (*C*_*h*_*/C*_*l*_) were calculated by means of presented model [Eqs. (5), (7–10) and (15)] and are shown in Fig. 6. Dependence of pressure difference across the membrane at which the voltage between electrodes in steady states is maximal (Δ*P*_max_) as a function of *C*_*h*_*/C*_*l*_ is shown in Fig. 6a for electrodes at distances from the membrane: 3 mm (curve 1), 5 mm (curve 2), and 7 mm (curve 3). In turn, maximal value of voltage between electrodes in steady states (Δ*ψ*_st_)_max_ as a function of initial quotient of KCl concentrations in chambers *C*_*h*_*/C*_*l*_ is presented in Fig. 6b for electrodes at distances 3 mm (curve 1), 5 mm (curve 2), and 7 mm (curve 3) from the membrane surfaces.

As results from Fig. 6, the height of the peak and pressure difference for maximal value of this peak depend on the location of electrodes near the membrane. For greater distance of electrodes from the membrane, the pressure (Δ*P*)_max_ is smaller for all *C*_*h*_/*C*_*l*_ (Fig. 6a). The maximal values of voltage in steady states (height of the peak, (Δ*ψ*_st_)_max_) are higher for electrodes located at a greater distance from the membrane (Fig. 6b). These dependencies are nonlinear; greater shifts of peak location and its maximal value are observed for higher *C*_*h*_/*C*_*l*_.

## Conclusions

1. As results from analysis of changes in time of activity of K^+^ ions near the membrane (Fig. 2) application of constant pressure difference across the membrane changes significantly the activity of K^+^ ions on both sides of the membrane. Greater activities of K^+^ ions are observed in the case when the mechanical pressure difference across the membrane (Δ*P*) is oriented with difference of osmotic pressure (greater activity of K^+^ ions for greater Δ*P*). In turn, fixing of mechanical pressure difference across the membrane directed in an opposite direction to osmotic pressure difference causes smaller activities of K^+^ ions to be observed (smaller activity of K^+^ ions for smaller Δ*P*). The above statements are also valid when the activity of K^+^ ions is measured at points at nonzero distances from the membrane. Changes in time of activities of K^+^ ions are lower when the points are more distant from the membrane. Such changes in the activity of K^+^ ions in the vicinity of the membrane are due to a disturbance of reconstruction of CBLs by mechanical pressure difference across the membrane.
2. The characteristic feature of voltage between electrodes in steady-states of the membrane system in concentration polarization conditions as a function of applied difference of mechanical pressure is the occurrence of a maximum. This maximum is observed in the range of difference of mechanical pressure directed in an opposite direction to difference of osmotic pressure (negative values of difference of mechanical pressure). An increase of difference of osmotic pressure across the membrane at initial moment causes a shift of observed peak toward higher (more negative) values of differences of mechanical pressure and an increase in the value of maximum. Furthermore, as results from Fig. 6, a shift of both electrodes toward the membrane causes a shift of the observed peak into higher values of pressure difference on the membrane accompanied by an increase of height of the peak.

## Appendix

The difference Eqs. (7–10) determine the behavior of K^+^ ions in CBLs and result from Eq. (6) for CBLs and the equation for flux of ions through the membrane (5). In order to transform the differential equation into difference equations (for CBL in chamber with lower concentration), we use

A1 $$ \frac{\partial C}{\partial t}\to \frac{C_{l,n}^{k+1}-{C}_{l,n}^k}{\varDelta t}, $$

A2 $$ \frac{\partial C}{\partial x}\to \frac{C_{l,n+1}^k-{C}_{l,n}^k}{d_w}, $$

A3 $$ \frac{\partial^2C}{\partial {x}^2}\to \frac{1}{d_w}\left(\frac{C_{l,n+1}^k-{C}_{l,n}^k}{d_w}-\frac{C_{l,n}^k-{C}_{l,n-1}^k}{d_w}\right)=\frac{1}{{d_w}^2}\left({C}_{l,n+1}^k-2{C}_{l,n}^k+{C}_{l,n-1}^k\right), $$

A4 $$ v={L}_p\varDelta p. $$

Inserting equations (A1-A4) into Eq. (6), we get

A5 $$ \frac{C_{l,n}^{k+1}-{C}_{l,n}^k}{\varDelta t}={D}_s\frac{1}{{d_w}^2}\left({C}_{l,n+1}^k-2{C}_{l,n}^k+{C}_{l,n-1}^k\right)-{L}_p\varDelta p\frac{C_{l,n+1}^k-{C}_{l,n}^k}{d_w} $$

Equation (A5) leads after simple transformations to Eq. (9). Analogously, we get Eq. (10) for CBL in the chamber with higher concentration.

In turn, for the first layer at the surface of the membrane in the chamber with a lower concentration (*l*), we use the equation

A7 $$ \frac{\partial C}{\partial t}=\frac{\varDelta J}{\varDelta x}\to \frac{J_{sm}-{J}_{1-2}}{d_w} $$

where *J*_*sm*_ is a flux of ions through a membrane defined by Eq. (5), while

A8 $$ {J}_{1-2}=-{D}_s\frac{\partial C}{\partial x}+vC $$

is the flux of ions through the surface between layers 1 and 2. Using equations (A1), (A2), (A4), (A8), and (5) in equation (A7) (for *n*=1 and $\frac{\partial C}{\partial t}\to \frac{C_{l,2}^k-{C}_{l,1}^k}{\varDelta t}$) we obtain Eq. (7) for the first layer in the chamber with lower concentration. Analogously, we get Eq. (8) for the first layer in chamber with higher concentration.

## Nomenclature

*J*_*v*_: Volume flux through the membrane
*J*_*s*_: Ion flux through the membrane
*ΔP* = *P*_*h*_ − *P*_*l*_: Mechanical pressure difference across the membrane
*Δπ*_*s*_ = *RT*(*C*_*h*_ − *C*_*l*_): Osmotic pressure difference across the membrane
${\overline{C}}_s=\left({C}_h-{C}_l\right){\left[ \ln \left({C}_h{C_l}^{-1}\right)\right]}^{-1}$: Average solute concentration in the membrane
*C*_*h*_ , *C*_*l*_: Solute concentrations in chambers under (*h* - higher concentration) and over (*l* - lower concentration) the membrane
(*C*_*l*_)_corr_: Corrected value of solute concentration in chamber with lower concentration (*C*_*l*_)
*C*_*i*_, *C*_*e*_: Solute concentrations at membrane surfaces in chamber with lower (*e*) and higher concentration (*i*) respectively
*C*_*i*_^*^, *C*_*e*_^*^: Solute concentrations at electrode surfaces in chamber with lower (*e*) and higher concentration (*i*) respectively
*C*_*l*,*n*_^*k*^, *C*_*h*,*n*_^*k*^: Concentrations in layers near membrane (*n* – layer number) in time *t = k*, in chambers with lower (*l*) or higher (*h*) solute concentration
*L*_*p*_: Hydraulic permeability coefficient of the membrane [m^3^ N^−1^ s^−1^]
*σ*_*s*_: Solute reflection coefficient
*ω*_*s*_: Solute permeability coefficient [mol N^−1^ s^−1^]
*D*_*s*_: Diffusion coefficient of solute in aqueous solution
*t*_*s*_: Transference number of solute *s*
*F*: Faraday number
*R*: Gas constant
*T*: Absolute temperature [K]
*d*_*w*_: Thickness of the layer
*S*: Membrane surface
**Δ***ψ*: Voltage between Ag|AgCl electrodes placed directly in solutions
